# Dynamic Transcriptomic Networks Underlying Early Bolting in Non-Heading Chinese Cabbage

**DOI:** 10.3390/plants15131982

**Published:** 2026-06-26

**Authors:** Xueqing Zhou, Liping Song, Liguang Tang, Meixiu Wu, Changbin Gao, Chunyu Zhang, Aihua Wang

**Affiliations:** 1 Wuhan Academy of Agricultural Sciences, Wuhan 430345, China; zhouxueqing21@163.com (X.Z.); lp19871120@126.com (L.S.); tangliguanghzau@163.com (L.T.); 2 National Key Laboratory of Crop Genetic Improvement, Huazhong Agricultural University, Wuhan 430070, China; wumx@webmail.hzau.edu.cn (M.W.); zhchy@mail.hzau.edu.cn (C.Z.)

**Keywords:** non-heading Chinese cabbage, early bolting, transcriptome, vernalization, *BrSOC1*

## Abstract

Bolting time is a pivotal agronomic trait that determines the yield and commercial quality of *Brassica rapa* ssp. *chinensis* var. *utilis*. To investigate the molecular basis of early bolting, an early-bolting line ‘m662’ and a late-bolting line ‘t151’ were used in this study. Phenotypic evaluation combined with shoot apical meristem (SAM) observation showed that 10 days of low-temperature vernalization markedly accelerated bolting in ‘t151’. Subsequently, SAM samples from ‘m662’, non-vernalized ‘t151’, and 10-day vernalized ‘V10-t151’ were collected at five developmental stages (7, 10, 13, 16, and 19 d after transplanting) for transcriptome sequencing. Weighted gene co-expression network analysis revealed that key module genes related to gibberellin signaling were specifically enriched in ‘m662’ before bolting, whereas those in the middle and late bolting stages were enriched in hormone response, cell cycle regulation, and floral organ development. In ‘t151’, hub genes detected at 7–13 d included three paralogs of the floral integrator gene *SOC1* and *BraA06.FPF1*. *BrSOC1* (*BraA03g024230.4C*) was significantly upregulated in response to vernalization. DEGs identified during the late developmental stage (16–19 d) included genes involved in transmembrane transport processes, flower development, reproductive shoot system development. Expression analysis across the three materials showed that vernalization accelerated bolting in ‘t151’ by repressing *BrFLC* expression and promoting *BrSOC1* expression. This study elucidates the dynamic transcriptomic network underlying early bolting in non-heading Chinese cabbage, providing key functional genes and mechanistic insights for bolting regulation and molecular breeding.

## 1. Introduction

*Brassica rapa* L. (2n = 20) is an important vegetable and oilseed crop belonging to the genus *Brassica* [1]. As the diploid progenitor (AA genome) of the *Brassica napus* (AACC) and *Brassica juncea* (AABB) in U’s triangle, *B. rapa* has undergone extensive diversification under agricultural selection during its relatively short domestication history, giving rise to multiple cultivated forms, including turnip (ssp. *rapa*), Chinese cabbage (ssp. *pekinensis*), non-heading Chinese cabbage (ssp. *chinensis*), and oilseed types (ssp. *oleifera*) [2]. Among these, non-heading Chinese cabbage represents the third most widely cultivated vegetable crop in China, with the planting area increasing from 0.534 million hectares in 2005 to approximately 1.534 million hectares in 2023 [3].

Based on morphological characteristics and utilization types, non-heading Chinese cabbage can be further classified into six varieties: pak-choi (var. *communis*), Tacai (var. *rosularis*), Caitai (var. *utilis*), Caixin (var. *parachinensis*), Taicai (var. *tai-tsai*), and Fenniecai cabbage (var. *multiceps*) [4,5]. Caitai (*Brassica campestris* ssp. *chinensis* var. *utilis*) as a flowering stalk vegetable exhibits exceptionally high nutritional and commercial value. Previous studies have shown that leaves, flowering stalks, and tender stems of *Brassica* vegetables are rich in glucosinolates, vitamin C, and carotenoids, conferring antioxidant, anti-inflammatory, and potential anticancer properties [6,7,8]. Untimely bolting leads to excessive consumption of nutrients, reducing both edible quality and commercial value, and ultimately causing yield loss [9]. In breeding progress, bolting and flowering times represent a key agronomic trait that directly determines yield, quality, and market value. Accordingly, systematic elucidation of the molecular basis underlying bolting in non-heading Chinese cabbage is crucial for modulating cultivar maturity and achieving synergistic enhancement of yield and quality.

Bolting and flowering represent a critical developmental transition from vegetative to reproductive growth, facilitating the completion of plant life cycles and reproductive development. Flowering time is a quantitative trait underpinned by complex genetic networks [10]. In *Arabidopsis thaliana*, approximately 300 genes have been reported to participate in flowering regulation, involving six major pathways, among which the vernalization pathway plays an important role by responding to prolonged low-temperature exposure [11]. Within this pathway, the *FRI*–*FLC* module constitutes the core regulatory unit. In late-bolting genotypes without vernalization, this module sustains high activity and profoundly inhibits floral transition. *FRIGIDA* (*FRI*), as an upstream regulator, interacts with transcriptional activation complexes to promote chromatin accessibility at the *FLOWERING LOCUS C* (*FLC*) locus, leading to elevated *FLC* transcription and the maintenance of late-flowering and strong vernalization-requiring phenotype [12]. *FLC* encodes a MADS-box transcriptional repressor that acts as a key flowering inhibitor in the vernalization pathway. It binds to the first intron of *FT* and the promoter region of *SOC1*, thereby repressing their transcription and inhibiting the transition of the shoot apical meristem (SAM) from vegetative to reproductive growth [13]. As a central component of the flowering network, *FT* encodes florigen, a mobile protein signal that transduces photoperiodic and temperature signals from leaves to the SAM and functions as a major positive regulator of flowering [14]. *SOC1* acts as a key integrator of multiple flowering pathways, including vernalization, photoperiod, and autonomous pathways, and operates synergistically with *FT* to promote floral initiation [15].

Although the molecular mechanisms governing vernalization pathways have been extensively characterized in *Arabidopsis*, distinct evolutionary features exist in *Brassica* species. These plants have experienced whole-genome triplication, which produces paralogs of key flowering regulators, including *FLC*, *FT* and *SOC1* with divergent expression patterns and biological functions [16]. In *Brassica rapa*, several *FLC* homologs have been identified, including *BrFLC1*, *BrFLC2*, *BrFLC3*, and *BrFLC5*, among which *BrFLC2* is considered the major regulator of bolting and flowering [17,18]. A natural mutation involving a deletion in the fourth exon of *BrFLC2* generates a premature stop codon, triggering nonsense-mediated mRNA decay (NMD) and leading to early flowering [19]. Furthermore, the expression level of *BrFLC2* is positively correlated with vernalization requirement, highlighting its central role in flowering repression [20]. Functional divergence among *FLC* paralogs has also been widely reported. *BrFLC1* and *BrFLC3* are more responsive to low-temperature signals, whereas *BrFLC2* plays a predominant role in maintaining stable repression of flowering [21]. In addition to the repression of *FLC* through epigenetic mechanisms, prolonged cold induces expression of vernalization-responsive factors such as *BrVIN3*, *BrVRN1*, and *BrVRN2*, which collaborate to modify chromatin states and stabilize transcriptional silencing of flowering repressors during vernalization [22]. In addition to the *FLC* module, flowering activators such as *FT* and the integrator *SOC1* also exhibit functional divergence in *Brassica*. *BrFT* paralogs are primarily expressed in leaves and function as mobile signals mediating long-distance communication to the SAM [23]. Their expression is negatively regulated by *BrFLC* and rapidly induced following vernalization to promote floral transition. Meanwhile, genetic assays demonstrated that *BrSOC1-1* and *BrSOC1-2* were sufficient to confer the late-bolting phenotype in Chinese cabbage [24]. However, studies on vernalization in *Brassica rapa* have been mostly limited to heading Chinese cabbage, while the underlying molecular mechanisms in non-heading Chinese cabbage remain poorly understood.

The shoot apical meristem (SAM) serves as a central regulator of plant development, giving rise to aerial organs such as leaves, stems, and flowers [25]. Therefore, systematically characterizing the morphological and molecular dynamics of the SAM during bolting is essential for understanding flowering regulation and improving crop production. In this study, an early-bolting line (m662) and a late-bolting line (t151) were used to investigate the regulatory effects of vernalization on bolting. By integrating bolting phenotypes, SAM histological observations, and *BrFLC2* expression profiles, we evaluated the response of the late-bolting line to vernalization and characterized the gene expression dynamics underlying the bolting transition in the early-bolting line. Furthermore, focusing on the SAM, we performed transcriptome sequencing at different time points combined with weighted gene co-expression network analysis (WGCNA) to systematically dissect the transcriptional reprogramming associated with vernalization-mediated SAM development and the transition from vegetative to reproductive growth. Key co-expression modules and hub genes were identified to elucidate the regulatory networks and expression patterns of vernalization-related genes. In summary, this study integrates phenotypic and transcriptomic profiling to uncover the molecular basis underlying bolting in non-heading Chinese cabbage, thereby filling an important knowledge gap and providing candidate genes for molecular breeding and precise control of flowering time.

## 2. Results

### 2.1. Bolting Process and Vernalization Response in Non-Heading Chinese Cabbage

**Figure 1:**
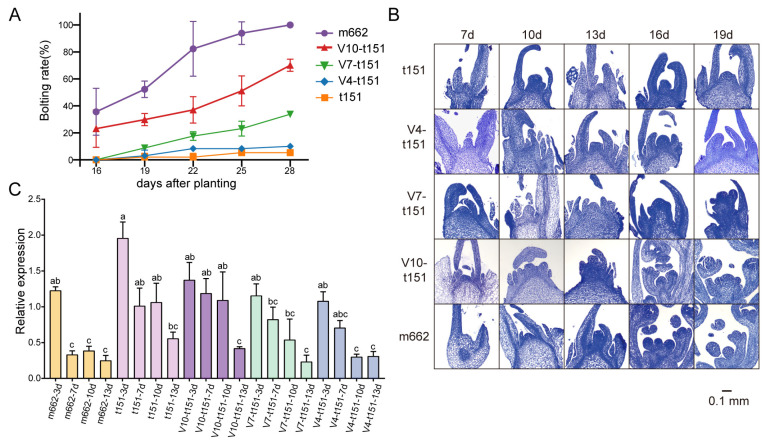
Bolting process and vernalization response in non-heading Chinese cabbage. (**A**) Bolting rate of five sample groups from 16 to 28 days after transplanting. Early-bolting line ‘m662’ and late-bolting line ‘t151’, ‘V4-t151’, ‘V7-t151’, and ‘V10-t151’ represent the ‘t151’ line subjected to 4, 7, and 10 days of vernalization pre-treatment, respectively. Each measurement was performed in three biological replicates, *n* = 50. (**B**) Shoot apical meristem longitudinal section comparison of early-bolting line and late-bolting line with or without vernalization, from 7 to 19 days after transplanting. Scale bar = 0.1 mm. (**C**) Expression levels of *BrFLC2* in leaf of early-bolting line and late-bolting line with or without vernalization, from 3 to 13 days after transplanting. Significant differences among groups were identified via one-way ANOVA combined with Tukey’s multiple range test. Different lowercase letters above the bars indicate significant differences among treatments at *p* < 0.05.

In *Arabidopsis thaliana*, flowering is coordinately regulated by multiple pathways, and whether a conserved flowering regulatory network exists in non-heading Chinese cabbage remains unclear. To investigate the effect of vernalization on the bolting process, we used the early-bolting line ‘m662’, the late-bolting line ‘t151’, and ‘t151’ subjected to different days of vernalization as experimental materials. The bolting ratio at different developmental stages (16–28 days) was recorded and analyzed (Figure 1A). Results showed that bolting differences among accessions were mainly detected at two critical stages. At 16–22 days after transplanting, the early-bolting line ‘m662’ was the first to complete the transition from vegetative to reproductive growth. At this stage, the bolting ratios of non-vernalized and short-term vernalized ‘t151’ lines were both below 40%, whereas the bolting ratio of ‘V10-t151’ (10 d vernalization) was higher than that of ‘t151’, ‘V4-t151’, and ‘V7-t151’. At 22–28 days after sowing, ‘m662’ completed bolting with a ratio reaching 100%. During this stage, ‘V10-t151’ rapidly initiated the transition to reproductive growth, showing a markedly increased bolting rate. The bolting ratio of ‘V7-t151’ was higher than that of ‘V4-t151’ but lower than that of ‘V10-t151’, exhibiting a gradient effect corresponding to vernalization duration. Taken together, low-temperature vernalization exerts a dose-dependent effect on the bolting ratio of ‘t151’, with a 10-day treatment significantly accelerating the transition to reproductive growth.

The morphological dynamics of the shoot apical meristem (SAM) provide a more precise indicator of the transition from vegetative to reproductive growth. Paraffin sectioning was employed to characterize SAM development across five experimental groups at 7, 10, 13, 16, and 19 d after transplanting, with a total of 25 samples (Figure 1B). Based on the developmental trajectory of SAM differentiation, the developmental period from 7 to 19 days could be divided into two distinct stages. During 7–13 days, the SAM of all materials gradually enlarged from an initially small protrusion into a more rounded structure, with primordium-like outgrowths forming at the periphery. By day 13, the early-bolting line ‘m662’ exhibited more prominent and elongated meristematic protrusions compared with the other materials. During 13–19 days, the SAM of ‘m662’ and ‘V10-t151’ progressively differentiated into floral meristems, continuously initiating floral primordia and completing the morphological transition from vegetative to reproductive growth. This process was accompanied by sustained primordium initiation and progressive pedicel elongation. In contrast, ‘t151’ and ‘V4-t151’ displayed a markedly delayed SAM developmental trajectory, characterized by a smooth and flattened apex with only conical leaf primordia at the periphery, thereby remaining in a vegetative state. ‘V7-t151’ exhibited an intermediate phenotype with SAMs showing slight enlargement and dome formation alongside 2–3 small primordia surrounding the meristem by day 19. Collectively, histological evidence indicates that ‘m662’ and ‘V10-t151’ complete the critical transition from vegetative to reproductive growth between 13 and 16 days, whereas SAM differentiation and bolting progression in the late-bolting line ‘t151’ are strongly modulated by vernalization.

To preliminarily investigate the regulatory effect of vernalization on the bolting process in the late-bolting line ‘t151’, leaf samples from five experimental groups were collected at 3, 7, 10, and 13 days after transplanting to analyze the expression profile of the core flowering repressor gene *BrFLC2* in the vernalization pathway [20] (Figure 1C, Table S1). qRT–PCR analysis showed that the expression level of *BrFLC2* exhibited an overall declining trend with developmental progression across all five groups, with significantly lower expression at day 7 compared with day 3 in all materials. Notably, the early-bolting line ‘m662’ maintained extremely low *BrFLC2* expression from 7 to 13 days, indicating that *BrFLC2* does not exert a repressive effect on flowering in this line. In contrast, the expression level of *BrFLC2* in non-vernalized ‘t151’ was higher than that in vernalized treatments, suggesting that vernalization suppresses the transcriptional accumulation of *BrFLC2*, thereby accelerating the bolting process in ‘t151’. Collectively, the integration of SAM histological observations and the expression dynamics of a key vernalization-related repressor gene reveals the morphological transition features and critical developmental time points underlying bolting in non-heading Chinese cabbage and provides a basis for subsequent dissection of its molecular regulatory mechanisms using transcriptome data at different time points.

### 2.2. RNA Sequencing and Dynamic Analysis of Differentially Expressed Genes

Previous results demonstrated that a 10-day vernalization treatment markedly promotes the bolting process in the late-bolting line ‘t151’, and that the critical transition from vegetative to reproductive growth in ‘m662’ and ‘V10-t151’ occurs between 7 and 19 days of development. Based on these findings, three materials, ‘m662’, ‘t151’, and 10-day vernalized ‘V10-t151’ were selected, and shoot apical meristem (SAM) samples were harvested at 7, 10, 13, 16, and 19 days after transplanting for transcriptome sequencing. The samples were designated as m7d, m10d, m13d, m16d, and m19d; t7d, t10d, t13d, t16d, and t19d; and V7d, V10d, V13d, V16d, and V19d, respectively (Table S2).

**Figure 2:**
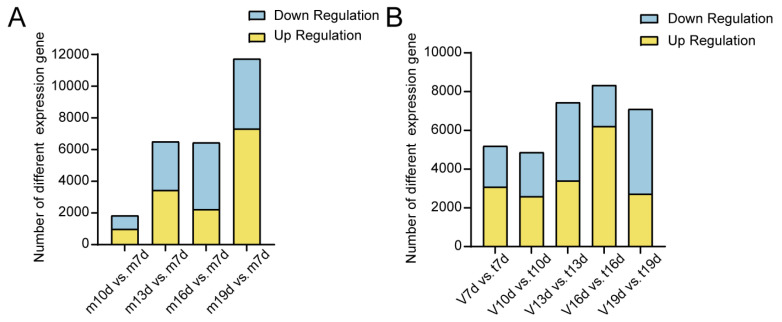
Numbers of differentially expressed genes (DEGs) among early and late-bolting materials at different SAM developmental stages. (**A**) Number of upregulated (yellow) and downregulated (blue) DEGs in m10d vs. m7d, m13d vs. m7d, m16d vs. m7d, and m19d vs. m7d. (**B**) Number of DEGs in V7d vs. t7d, V10d vs. t10d, V13d vs. t13d, V16d vs. t16d, and V19d vs. t19d.

Pearson correlation analysis further indicated that correlation coefficients among biological replicates of the 45 samples were all greater than 0.99, demonstrating reliability of the transcriptome data (Figure S1A). Principal component analysis (PCA) showed that biological replicates clustered tightly within each group, and samples from different developmental stages exhibited clear separation patterns (Figure S1B). Based on the high-quality transcriptome dataset, two differential expression comparison strategies were established for subsequent analyses (Table S3). In the first strategy, the day 7 sample of ‘m662’ (m7d) was used as the control to identify differentially expressed genes (DEGs) across developmental stages (m10d vs. m7d, m13d vs. m7d, m16d vs. m7d and m19d vs. m7d). The results showed that, relative to m7d, the number of DEGs at m13d was higher than that at m10d, indicating a progressive increase during development. The number of DEGs remained relatively stable between m13d and m16d, followed by a pronounced increase at 19 days, reaching the highest level, with 7315 upregulated and 4416 downregulated genes. These findings indicate that ‘m662’ undergoes extensive transcriptional reprogramming between 16 and 19 days, suggesting that a large gene set is involved in regulating the transition from vegetative to reproductive growth (Figure 2A). In the second strategy, samples of ‘t151’ at corresponding developmental stages were used as controls to identify DEGs in ‘V10-t151’. The number of DEGs at 7, 10, and 13 days was 2660, 1885, and 3339, respectively. At 16 and 19 days, the number of DEGs increased substantially to 8234 and 9640, respectively, indicating that the transcriptional impact of low-temperature vernalization on ‘t151’ was most prominent during 16–19 days of development (Figure 2B).

### 2.3. Co-Expression Module and Core Gene Analysis During Bolting in Early-Bolting Non-Heading Chinese Cabbage

**Figure 3:**
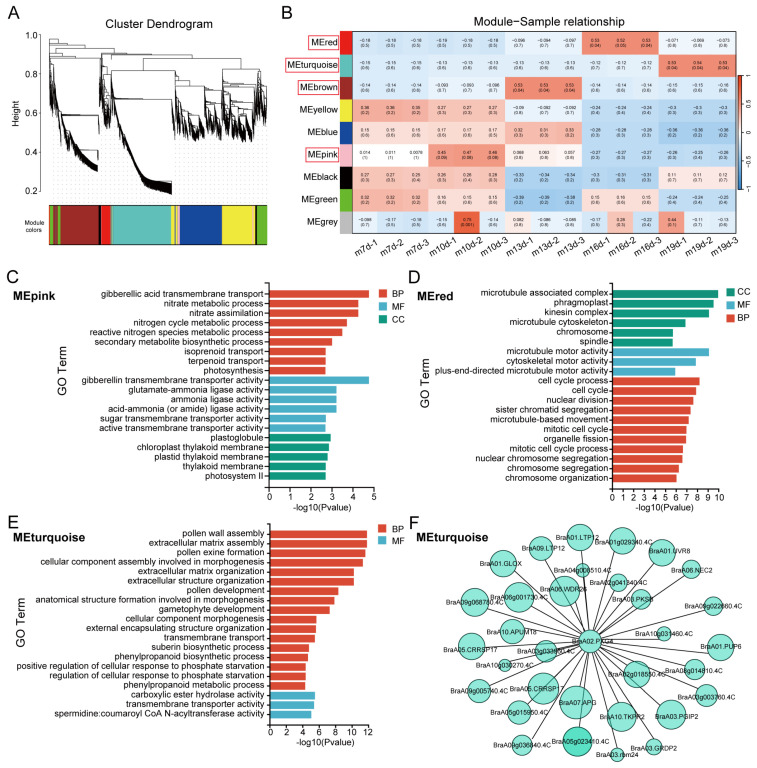
Weighted gene co-expression network analysis of differentially expressed genes in ‘m662’. (**A**) Hierarchical clustering dendrogram showing co-expression modules. Each leaf represents a gene, and genes were clustered based on a dissimilarity measure. Modules are indicated by the color bars below the dendrogram. (**B**) Module–trait relationships. Red and blue indicate positive and negative correlations, respectively, with correlation coefficients and *p*-values shown. (**C**) GO enrichment analysis of genes in the MEpink module. (**D**) GO enrichment analysis of genes in the MEred module. (**E**) GO enrichment analysis of genes in the MEturquoise module. (**F**) Co-expression network of hub genes in the MEturquoise module.

Plant growth and developmental transitions are accompanied by complex transcriptional regulation and extensive transcriptomic reprogramming. To investigate gene expression patterns before and after bolting and identify key hub genes in the early-bolting non-heading Chinese cabbage, weighted gene co-expression network analysis (WGCNA) was performed. Using the early-bolting line ‘m662’ as the material, lowly expressed differentially expressed genes (DEGs) (FPKM < 1) were filtered out, which resulted in a final set of 10,808 DEGs for downstream analyses. A scale-free co-expression network was then constructed, and DEGs were partitioned into nine co-expression modules, each representing a cluster of genes with highly similar expression trajectories (Figure 3A). Module–trait correlation analysis revealed clear time-specific associations between co-expression modules and developmental stages. In particular, the MEpink module showed a moderate positive correlation with the 10-day samples (correlation coefficient = 0.46). The MEbrown, MEred, and MEturquoise modules were strongly positively correlated with the 13-day, 16-day, and 19-day samples, respectively, with an average correlation coefficient of 0.53 (*p* < 0.05) (Figure 3B). Notably, genes within these modules were predominantly associated with a single developmental stage, indicating pronounced stage-specific expression patterns over time. These genes may play pivotal regulatory roles at distinct time points during the transition from vegetative to reproductive growth in non-heading Chinese cabbage. Gene Ontology (GO) enrichment analysis was performed on these three core modules to elucidate the functional roles of key genes at different developmental stages.

MEpink module comprised 185 genes and showed a high correlation with the 10-day samples. GO enrichment analysis revealed that this module was enriched across all three categories: biological process (BP), molecular function (MF), and cellular component (CC). The most significant terms were gibberellic acid transmembrane transport (BP) and gibberellin transmembrane transporter activity (MF), indicating that gibberellin-related processes play a key role during this developmental stage (Figure 3C, Table S4). To further identify key regulatory genes within this module, a co-expression network was constructed based on highly weighted genes, and two hub genes were identified, *BraA02g045780.4C* and *BraA03o63130.4C*. *BraA02g045780.4C* is associated with cell wall remodeling, whereas *BraA03g063130.4C* encodes an enzyme involved in the synthesis of activated sulfate (Figure S2A). MEbrown module comprised 2086 genes and showed a significant correlation with the 13-day samples. GO enrichment analysis indicated that this module was predominantly enriched in the BP category. The most significantly enriched GO terms included response to oxygen-containing compounds, response to hormones, and response to chitin (Figure S2B, Table S5), suggesting that stress-related and hormone-mediated regulatory processes are active at this developmental stage. Three core genes were identified, including *BraA08g018970.4C* encoding xyloglucan endotransglucosylase/hydrolase 24 (XTH24), *BraA06g027310.4C* encoding the NAC062 transcription factor, and *BraA05g039950.4C* encoding the protein kinase ATK2. These hub genes are mainly involved in cell wall remodeling and intracellular signal transduction processes (Figure S2C). MEred module comprised 511 genes and was significantly associated with the 16-day samples. GO enrichment analysis showed that this module was enriched across all three categories (Figure 3D, Table S6). In the CC category, genes were enriched in microtubule-associated complex and phragmoplast. In the MF category, enrichment was mainly in microtubule motor activity and cytoskeletal motor activity. In the BP category, genes were significantly enriched in cell cycle-related processes, including cell cycle process, nuclear division, and sister chromatid segregation. These results indicate that the MEred module is closely associated with cell proliferation and cytoskeletal dynamics in the shoot apical meristem. A flowering-related hub gene, *BraA08g001310.4C*, encoding a GDSL-type esterase/lipase (GELP), was identified based on the co-expression network (Figure S2D). The MEturquoise module comprised 2958 genes and showed a strong association with the 19-day samples. GO enrichment analysis indicated that this module was predominantly enriched in the BP category, with the most significantly overrepresented terms related to pollen development, including pollen wall assembly, pollen exine formation, and pollen development (Figure 3E, Table S7). *BraA02g02660.4C* was identified as a core gene encoding PXG4, which acts as a negative regulator in the abscisic acid (ABA) signaling pathway (Figure 3F). Collectively, these four time-specific co-expression modules reveal dynamic changes in the transcriptional regulatory network underlying the transition from vegetative to reproductive growth in early-bolting non-heading Chinese cabbage: the 10-day stage emphasizes gibberellin signaling, the 13-day stage is dominated by hormone and stress responses, the 16-day stage reflects cell proliferation and cytoskeletal dynamics, and the 19-day stage highlights pollen development and ABA signaling. These results provide a valuable dataset for constructing a gene regulatory network of bolting development and lay a theoretical foundation for further elucidation of the molecular mechanisms controlling early bolting traits.

### 2.4. Co-Expression Network Analysis of Vernalization-Regulated Bolting in Non-Heading Chinese Cabbage

**Figure 4:**
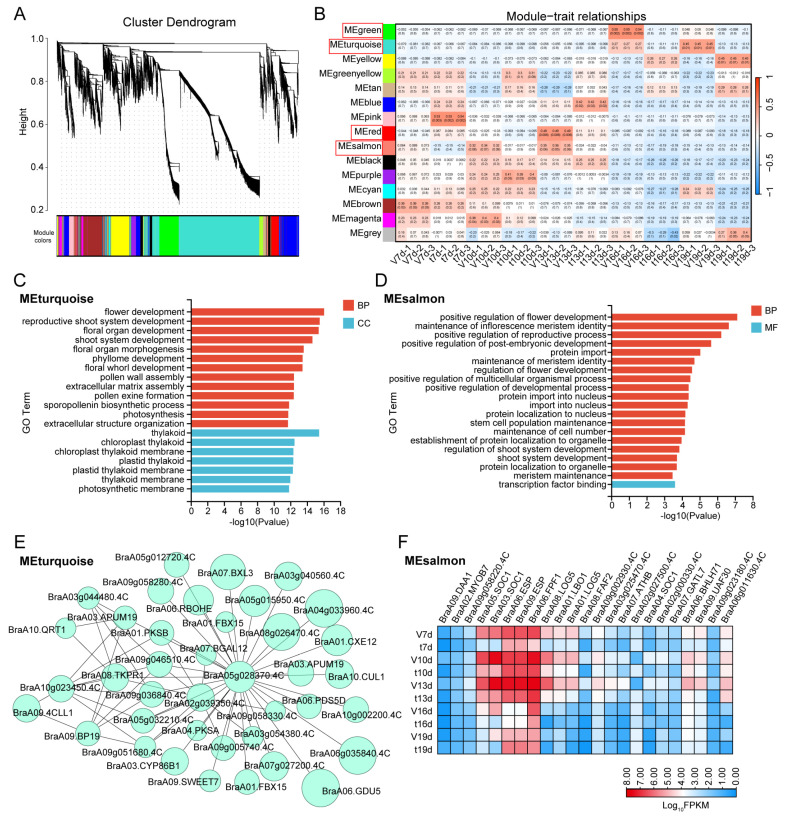
Weighted gene co-expression network analysis of differentially expressed genes in ‘t151’ with or without vernalization. (**A**) Hierarchical clustering dendrogram showing co-expression modules. Each leaf represents a gene, and genes were clustered based on a dissimilarity measure. Modules are indicated by the color bars below the dendrogram. (**B**) Module–trait relationships. Red and blue indicate positive and negative correlations, respectively, with correlation coefficients and *p*-values shown. (**C**) GO enrichment analysis of genes in the MEturquoise module. (**D**) GO enrichment analysis of genes in the MEsalmon module. (**E**) Co-expression network of hub genes in the MEturquoise module. (**F**) Heatmap of genes in the MEsalmon module.

To elucidate the molecular mechanisms underlying vernalization-mediated regulation of bolting in non-heading Chinese cabbage, WGCNA was performed using the DEGs previously identified in ‘V10-t151’. After filtering out lowly expressed genes (FPKM < 1), a total of 8263 DEGs were retained and classified into 15 co-expression modules based on temporal expression patterns (Figure 4A). Module–trait correlation analysis revealed that the MEgreen and MEturquoise modules were significantly positively correlated with the 16-day and 19-day samples of ‘V10-t151’, with correlation coefficients of 0.55 and 0.45, respectively (Figure 4B).

The MEgreen module comprised 638 genes. GO enrichment analysis indicated that genes in this module were mainly involved in transmembrane transport processes, with significant enrichment in pathways related to the transport of organic acids, carboxylic acids, and neutral amino acids (Figure S3A, Table S8). In addition, BP category was mainly enriched in fatty acid metabolism, monocarboxylic acid metabolism, and lipid metabolic pathways. The MEturquoise module contained 2832 genes and showed significant enrichment in BP associated with floral organ formation and shoot apical meristem development (Figure 4C, Table S9). GO analysis revealed significant enrichment in flower development, reproductive shoot system development, shoot system development, and photosynthesis. At the CC category, genes in this module were mainly associated with chloroplast thylakoid and photosynthetic membrane components, indicating a close link to photosynthetic activity and developmental regulation. A core co-expression network was constructed based on gene connectivity. Several key hub genes were identified, including *BraA10g023450.4C* encoding a glycoside hydrolase, as well as two candidate regulatory genes with unknown functions, *BraA05g023450.4C* and *BraA02g039350.4C* (Figure 4E).

Notably, the MEsalmon module showed positive correlations with the 7-, 10-, and 13-day samples, with the highest association observed at 13 days (correlation coefficient = 0.35). This module contained only 23 genes. GO enrichment analysis revealed significantly enriched pathways related to bolting and floral development, including positive regulation of flower development and regulation of shoot system development (Figure 4D, Table S10). Within this module, three *SOC1* homologs were identified: *BraA03g024230.4C*, *BraA04g033900.4C*, and *BraA05g005540.4C*. Additional key genes were identified, including the flowering activator *BraA06.FPF1* (Figure 4F). During the 7–13-day developmental stage, the expression levels of *SOC1* family genes in ‘V10-t151’ were significantly higher than those in the non-vernalized control ‘t151’. In addition, the MEred module showed a correlation coefficient of 0.49 with the 13-day samples. GO enrichment analysis indicated that this module was mainly involved in stress- and signal-response processes, including response to chitin (Figure S3).

Because gibberellin signaling was observed to play a key role before bolting in the early-bolting line, we investigated whether a similar mechanism occurs in vernalized ‘10V-t151’. Using the 7-day sample as a baseline, 8796 differentially expressed genes across the subsequent four time points were subjected to WGCNA (Figure S4). GO enrichment analysis of the genes within the highly correlated modules at 10 and 13 days revealed that gibberellin signaling did not play a major regulatory role in ‘10V-t151’, indicating that this pathway is specific to the early-bolting line ‘m662’. Collectively, low-temperature vernalization promotes the transition from vegetative to reproductive growth in the late-bolting line ‘t151’ by regulating multiple biological processes, including metabolic pathways, cellular development, floral organ formation, and coordinated transcriptional regulatory networks associated with bolting, thereby accelerating the bolting process.

### 2.5. Expression Pattern Analysis of Key Vernalization Pathway Genes

**Figure 5:**
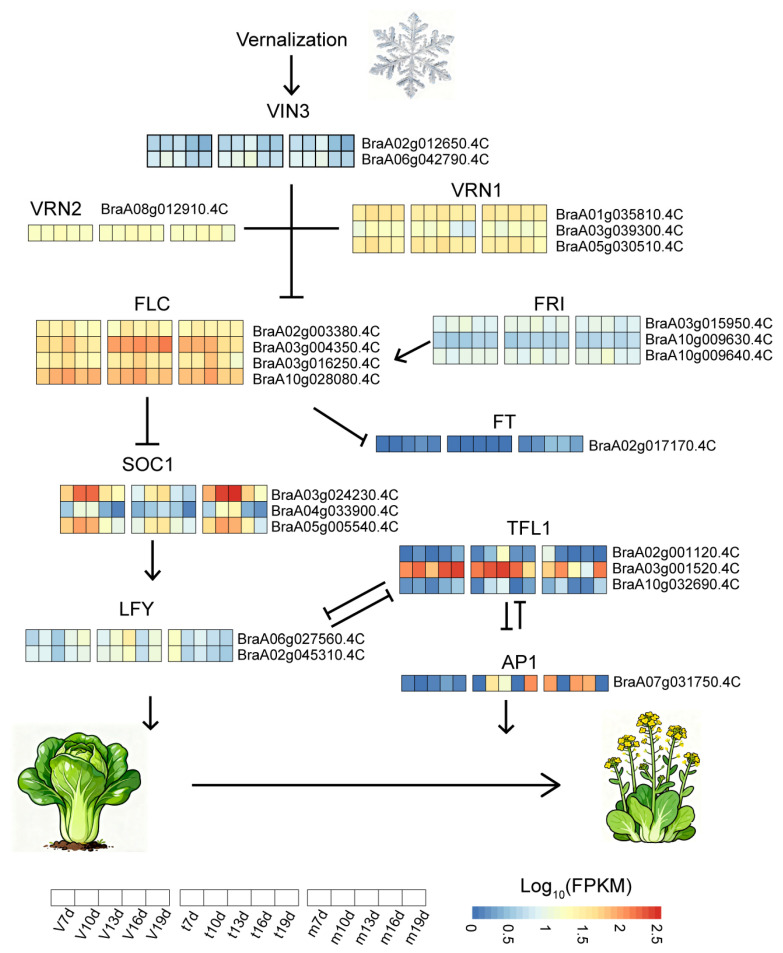
Expression patterns of key genes in the vernalization pathway. The color scale represents gene expression levels based on Log_10_(FPKM) values. Three sample groups, each with five developmental stages. From left to right, the blocks represent V7d, V10d, V13d, V16d, V19d; t7d, t10d, t13d, t16d, t19d; and m7d, m10d, m13d, m16d, m19d. M, t, and V represent m662, t151, and t151 with 10 days of vernalization, from 7 to 19 days after transplanting, respectively.

The key regulatory genes and mechanisms of the vernalization pathway have been extensively studied in *Arabidopsis*. To understand the expression patterns of core vernalization pathway genes in non-heading Chinese cabbage, we systematically identified and analyzed the transcriptional expression profiles of these key genes across different materials and developmental stages (Figure 5).

*FLC* is a core floral repressing gene in the vernalization pathway, which negatively regulates the transition to flowering [19]. In this study, four *Brassica rapa FLC* homologs were identified (*BraA02g003380.4C*, *BraA03g004350.4C*, *BraA03g016250.4C*, and *BraA10g028080.4C*). Among them, *BraA03g004350.4C* was upregulated in the late-bolting line ‘t151’, with expression levels much higher than those in the early-bolting material ‘m662’ and the vernalized ‘V10-t151’. Meanwhile, we identified three *BrSOC1* homologs (*BraA03g024230.4C*, *BraA04g033900.4C*, and *BraA05g005540.4C*). Among them, *BraA03g024230.4C* exhibited significantly lower expression at 10 and 13 days in ‘t151’ compared to ‘V10-t151’ and ‘m662’, further confirming that vernalization treatment relieves *BrFLC* repression and promotes *BrSOC1* upregulation. Additionally, in the early-bolting material ‘m662’, the expression of *BrFT* (*BraA02g017170.4C*) continuously increased during development, positively driving early bolting and flowering. Three homologs of the floral repressor *TFL1* were identified in this study. Among them, *BraA03g001520.4C* exhibited significantly higher expression levels in ‘t151’ and ‘V10-t151’ compared to ‘m662’; it may delay bolting in late-bolting materials by inhibiting floral meristem formation. Furthermore, the vernalization-specific inducer *VIN3*, the flowering repressor *FRI,* and the floral meristem gene *LFY* consistently exhibited low expression levels across all three experimental materials. The key vernalization genes *VRN2* and *VRN1*, along with the floral meristem determinant *AP1*, did not display any consistent differential expression patterns across the materials or developmental stages. These findings indicate that *BrFLC* and *BrSOC1* act as core regulators of differential bolting performance in non-heading Chinese cabbage.

### 2.6. qRT-PCR Validation of Transcriptome Data

**Figure 6:**
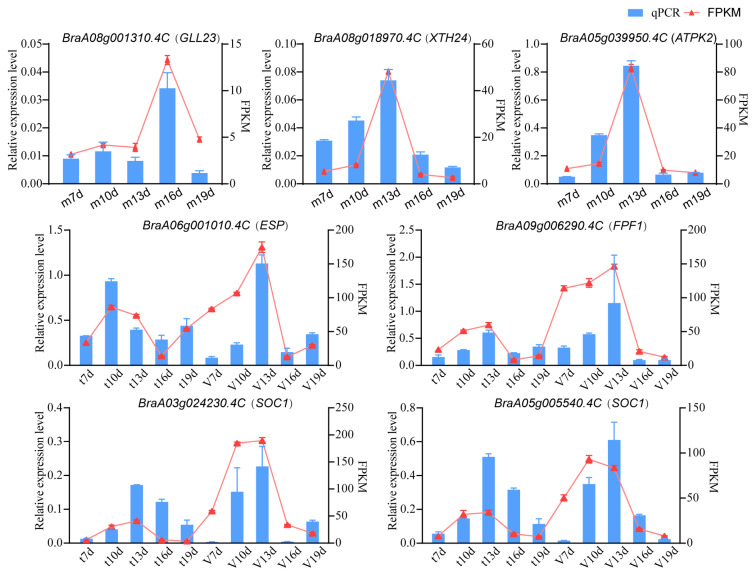
qRT-PCR validation of expression patterns of three DEGs in ‘m662’, and four DEGs in ‘t151’ and ‘V10-t151’.

To verify the reliability of the transcriptome sequencing data and the accuracy of differentially expressed gene levels, we selected several core genes identified through WGCNA for expression validation using quantitative real-time PCR (qRT-PCR) (Figure 6, Table S1). For the verification experiments, three core genes, *BraA08g001310.4C*, *BraA08g018970.4C*, and *BraA05g039950.4C*, were selected for samples from early-bolting material ‘m662’. For the late-bolting material ‘t151’ and the vernalized material ‘V10-t151’, four core genes, *BraA06g001010.4C*, *BraA09g006290.4C*, *BraA03g024230.4C*, and *BraA05g005540.4C*, were chosen for validation. The qRT-PCR validation results showed a strong correlation with the transcriptome sequencing (mRNA-Seq) data, with both the expression patterns and the trends in differential expression aligning closely. The trends in differential gene expression observed by qRT-PCR closely matched those identified in the transcriptomic analysis, further validating the accuracy and reliability of the sequencing data.

## 3. Discussion

This study investigates the bolting traits of two different genotypes of non-heading Chinese cabbage, focusing on how early bolting occurs in one genotype and how vernalization regulates bolting in the other. By integrating bolting phenotypes, shoot apical meristem (SAM) development, and transcriptomic data collected at multiple developmental stages, we systematically explored the molecular mechanisms underlying bolting in these materials. Our results demonstrate that low-temperature vernalization significantly accelerates bolting in the late-bolting line ‘t151’, with its effect being duration-dependent. Transcriptomic analysis further indicated that this transition is accompanied by stage-specific transcriptional reprogramming. Using co-expression network analysis, we identified key modules and hub genes associated with different developmental stages. Together with the expression patterns of key vernalization pathway genes, our study provides new insights into the *BrFLC*-*BrSOC1*-centered bolting and flowering regulation module in non-heading Chinese cabbage, highlighting the role of gibberellin signaling and emphasizing the coordination between metabolic and developmental pathways. This research lays the foundation for a comprehensive molecular framework to understand bolting regulation in Chinese cabbage.

### 3.1. Mechanistic Insights into Early Bolting Regulation in Non-Heading Chinese Cabbage

In this study, the early-bolting line ‘m662’ was subjected to differential expression analysis using its early developmental stage (7 d) as reference. The DEGs identified reflect the dynamic transcriptional changes within the same genotype during development, revealing the regulatory trajectory of endogenous developmental programs underlying bolting. Results from WGCNA revealed distinct temporal specificity among co-expression modules across different developmental stages. At the early developmental stage (10 d), the gibberellin pathway was highly enriched. Subsequently, at 13 d, differentially expressed genes were predominantly enriched in pathways associated with hormone responses and environmental signal transduction, indicating that plants had completed the integration of internal signals prior to morphological transition, thereby laying a molecular foundation for the subsequent floral transition. These observations are highly consistent with previous studies demonstrating that plants undergo a critical phase of signal integration prior to the initiation of floral transition [26]. As development proceeded to 16 d, significant enrichment was observed in genes involved in the cell cycle and microtubule-related pathways, indicating that the shoot apical meristem entered a phase of rapid cell division and structural reorganization. The indispensable roles of the microtubule cytoskeleton in cell division and organogenesis have been extensively documented [27]. Moreover, the regulation of meristem size and cell proliferation rate is considered a key determinant in controlling the timing of developmental transitions [28]. Therefore, this stage represents the critical cytological basis for the transformation of the shoot apical meristem into the floral meristem. At the late developmental stage (19 d), differentially expressed genes were significantly enriched in floral organ formation and pollen development, indicating that the plant had completed the transition to reproductive growth. Collectively, the transcriptomic regulation of ‘m662’ follows a typical time-driven cascade model, progressing sequentially from signal integration to structural remodeling and ultimately achieving organ differentiation. Genes related to both the vernalization pathway and gibberellin pathway may contribute substantially to its early-bolting phenotype. Such sequential transcriptional reprogramming suggests that the early-bolting characteristic of ‘m662’ is primarily attributed to the specificity of its genetic background, which enables the precocious initiation and rapid progression of developmental programs. These findings provide a mechanistic explanation for how early-bolting materials achieve accelerated bolting and identify the critical developmental stages involved.

### 3.2. Molecular Basis of Vernalization-Promoted Bolting in Late-Bolting Materials

In contrast to ‘m662’, the analysis of the late-bolting line ‘t151’ was performed using a comparative strategy in which non-vernalized plants at the same developmental stage served as the control, and vernalized plants served as the treatment group. Accordingly, the identified differentially expressed genes reflect the progressive amplification of vernalization signals at various developmental stages of the plant following seed vernalization. WGCNA revealed that vernalization induced substantial transcriptional responses at all developmental stages. During early development (7–13 d), the module containing the *BrSOC1* homolog was activated in vernalized plants. As a central integrator in the flowering regulatory network, upregulation of *SOC1* marks the initiation of the floral transition program [29]. Low temperature suppresses *FLC* expression through epigenetic modifications, thereby releasing its transcriptional repression on *SOC1* and *FT* [30,31]. Therefore, the precocious activation of *BrSOC1* in ‘V10-t151’ indicates that vernalization has triggered the flowering signaling pathway at an early stage. The most extensive network restructuring occurred from 16 to 19 d. During this period, modules significantly correlated with ‘V10-t151’ were mainly enriched in pathways including lipid metabolism, amino acid metabolism, and floral organ development. These results indicate that vernalization promotes reproductive growth not only by regulating flowering signaling pathways but also by reprogramming primary metabolic processes to supply sufficient materials and energy. Previous studies have demonstrated that metabolic signals can promote floral transition by interacting with the flowering regulatory network [32,33], and we observed similar patterns in non-heading Chinese cabbage.

### 3.3. Cross-Genotype Analysis of Hub Genes and Floral Regulators

Distinct differential expression analysis strategies were adopted for the two lines in this study, with their transcriptional regulatory features corresponding to biological processes at distinct levels. ‘M662’ reflects the temporal progression of endogenous developmental programs, while ‘t151’ exhibits transcriptional network remodeling in response to vernalization signals. Expression profiles of core vernalization-related genes clarify the developmental divergence between the two genotypes. In ‘t151’, sustained high expression of *FLC* homologs continually represses downstream flowering genes. Vernalization reduces *FLC* expression through epigenetic mechanisms, thereby relieving its repression on *SOC1* and *FT* and triggering systematic restructuring of the transcriptional network [30]. In contrast, the inhibitory effect of this pathway is weaker in ‘m662’, allowing it to bypass vernalization and directly initiate developmental programs, possibly because *FLC* in ‘m662’ lacks a functional role in repressing flowering. The high expression of this *BrFLC* may be a key molecular factor contributing to the late bolting characteristic of ‘t151’. *FLC* inhibits the expression of downstream flowering integrator genes, *FT* and *SOC1*, through transcriptional repression, thereby delaying the transition from vegetative to reproductive growth [13]. Notably, the expression variation range of *BrSOC1* was obviously higher than that of *BrFLC* in this study. This phenomenon may reflect a signal amplification pattern within the flowering regulatory network. Multiple copies of *BrFLC* widely exist in Brassica plants and can reduce their expression fluctuation to a certain extent. As a critical hub integrating multiple signaling pathways, *SOC1* expression is controlled not only by *FLC* but also jointly regulated by photoperiod metabolism and hormone signals. This complex regulatory mode makes *SOC1* more likely to produce obvious expression differences [29]. Relevant studies on shoot apical meristem regulation have drawn similar conclusions [34]. Downstream integration genes usually show more sensitive expression changes than upstream repressive genes. By distinguishing between two types of transcriptional changes (temporal dynamics and treatment responses), this study reveals the molecular basis of bolting in non-heading Chinese cabbage with different genotypes at the network level and provides a new perspective for deciphering the vernalization-dependent flowering regulatory mechanism.

Collectively, the present findings illustrate that vernalization signals initiate developmental programs by relieving key inhibitory effects during the bolting process of non-heading Chinese cabbage. Hormone signaling, cell proliferation regulation and metabolic pathways function synergistically at distinct stages to jointly drive structural remodeling and organ differentiation in the shoot apical meristem. This multi-pathway coordinated regulatory model enables plants to precisely adjust flowering time under complex environmental conditions. Based on WGCNA results, several candidate key genes were identified across different developmental stages in this study. These genes exhibit high connectivity within the co-expression network and may serve essential functions in bolting regulation of non-heading Chinese cabbage. In the early bolting line ‘m662’, the hub gene *XTH24* (*BraA08g018970.4C*) was identified from the MEbrown module. It belongs to the xyloglucan endotransglucosylase/hydrolase (XTH) family. Enzymes of this family are essential for cell wall remodeling and participate in organ elongation and developmental regulation in plants [35]. NAC transcription factors have been proven to control organ formation, cell division and cell differentiation [36]. This suggests that *NAC062* potentially modulates bolting transition through the regulation of downstream developmental genes. The GDSL-type esterase/lipase (GELP) gene *BraA08g001310.4C* was detected in the MEred module. GDSL proteins are widespread across plant species and engage in several biological processes, including growth, organ differentiation and pollen development [37]. These proteins are tightly associated with cell wall modification and lipid metabolism, and modulate cell division and tissue formation [38], which is consistent with the enrichment of cell cycle and microtubule-related pathways in this module. Moreover, gibberellin signaling was observed to play a key role before bolting in the early-bolting line ‘m662’, whereas no similar effect was detected in vernalized ‘t151’, providing a mechanistic explanation for the earlier bolting of ‘m662’ relative to vernalized ‘t151’.

Overall, the candidate key genes identified in this study are involved in cell wall remodeling, transcriptional regulation, cellular structure modulation and metabolic processes, revealing the complexity of the bolting regulatory network in non-heading Chinese cabbage. These genes provide valuable clues for exploring the molecular mechanisms underlying bolting and offer promising targets for further functional validation and molecular breeding. Although phenotypic investigation, transcriptome and co-expression network analysis systematically clarified the molecular regulatory characteristics of bolting in non-heading Chinese cabbage, certain limitations still exist. First, all analyses in this work rely on transcriptomic data, without verification from protein interaction assays. Second, the hub genes screened via WGCNA are determined based on expression correlation, and their exact roles in bolting control need to be further verified through gene editing experiment. Moreover, this study only focused on the effects of 10-day vernalization on bolting, while the dynamic changes in regulatory networks under different vernalization durations and temperature regimes remain poorly understood. Previous studies have demonstrated that vernalization exhibits an obvious accumulated temperature effect, and varied durations of low-temperature exposure can lead to gradient changes in flowering time [30]. Future research will focus on the core co-expression modules and candidate genes screened herein. Combined with genetic mapping and omics data, further efforts will be made to dissect the genetic and molecular mechanisms by which vernalization signals regulate SAM developmental transition. Functional verification via gene editing will be conducted to clarify the regulatory roles of key genes during bolting. In addition, molecular markers will be developed based on hub genes and their elite allelic variations, to support the molecular design breeding of non-heading Chinese cabbage varieties with different maturity traits.

## 4. Materials and Methods

### 4.1. Plant Materials and Sample Collection

To explore the bolting traits of non-heading Chinese cabbage, two inbred lines, the early-bolting line ‘m662’ and late-bolting line ‘t151’, were used as experimental materials. Both lines have been self-purified for continuous years with highly homozygous genetic backgrounds. Seeds with uniform growth status were selected for germination on moist filter paper. For vernalization pretreatment, seedlings of ‘t151’ were cultivated at 4 °C under a light intensity of 200 μmol·m^−2^·s^−1^ with a 16 h/8 h light/dark photoperiod. Three vernalization durations were set, including 4 d (V4-t151), 7 d (V7-t151) and 10 d (V10-t151). Non-vernalized ‘m662’ and ‘t151’ were established as control groups. All seedlings were transplanted into substrate soil and cultivated at a constant temperature of 24 °C, light intensity of 320 μmol·m^−2^·s^−1^, and a 16 h/8 h light/dark photoperiod. The day of transplantation was designated as day 1 of SAM development. Transplantation of all materials was completed on the same day.

A total of 60 leaf samples were collected, covering five groups (V4-t151, V7-t151, V10-t151, m662 and t151), four time points (3, 7, 10 and 13 d), with three biological replicates per treatment, to detect the expression level of *BrFLC2*. A total of 45 shoot apical meristem samples were additionally harvested for transcriptome sequencing, including three lines (V10-t151, m662 and t151), five time points (7, 10, 13, 16 and 19 d) and three biological replicates for each group. Each sample consisted of approximately 100 mixed shoot apical meristems.

### 4.2. Paraffin Section

Shoot apical meristems were collected from no less than three non-heading Chinese cabbage plants. Samples were immediately fixed in FAA solution (Servicebio, Wuhan, China) at 4 °C with a tissue-to-fixative volume ratio of 1:10. Tissue embedding was performed according to a previously described method [39], and embedded samples were stored at 4 °C. The trimmed trapezoidal paraffin blocks were cut into continuous sections of 5–7 μm using a microtome. Sections were flattened in 50% ethanol, unfolded in a water bath at 50 °C, mounted on slides, and dried at 37 °C for 24 h. All sections were deparaffinized in xylene three times for 10 min each, followed by rehydration through a graded ethanol series from 100% to 50%. The sections were stained with 0.5% toluidine blue, dehydrated with gradient ethanol, cleared in xylene, and sealed with neutral balsam. Microscopic observation and image capture were conducted using a LEICA DM750 microscope (Leica Microsystems, Wetzlar, Germany). According to previously described criteria [40], bolting was defined as stalk elongation of at least 0.5 mm or the appearance of visible flower buds. The transition from vegetative to reproductive growth was considered complete when the shoot apical meristem (SAM) progressed from growth cone elongation during floral bud differentiation to the initial emergence of floral primordia.

### 4.3. RNA Extraction and Quantitative Real-Time PCR Analysis

Total RNA was isolated from samples using the Eastep Super Total RNA Extraction Kit (Promega Biotech Co., Ltd., Beijing, China). First-strand cDNA was synthesized from 2 μg of total RNA using the RevertAid First Strand cDNA Synthesis Kit (Vazyme Biotech Co., Ltd., Nanjing, China) according to the manufacturer’s instructions. Quantitative real-time PCR (qRT-PCR) was performed on a CFX384 Touch Real-Time PCR Detection System (Bio-Rad Laboratories, Shanghai, China) using ChamQ Universal SYBR qPCR Master Mix (Vazyme). The sequences of the qRT-PCR primers are listed in Table S1. *BraA03g014820.4C* was used as the internal reference gene for normalization. Relative gene expression levels were calculated using the 2^−ΔΔCt^ method, where ΔCt was obtained by subtracting the Ct value of the reference gene from that of the target gene, and ΔΔCt was calculated relative to the designated control sample. Each sample was analyzed with three biological replicates and three technical replicates. Data were visualized using GraphPad Prism version 9.0 (GraphPad Software, Boston, MA, USA). Relative expression data were subjected to one-way analysis of variance (ANOVA) followed by Tukey’s multiple comparison test. Differences among means were considered statistically significant at *p* < 0.05, and different lowercase letters were used to indicate significant differences among treatments.

### 4.4. Transcriptome Sequencing and Differentially Expressed Gene Analysis

RNA samples were sent to Shanghai Personal Biotechnology Co., Ltd. (Shanghai, China) for library construction and sequencing on the Illumina X Plus platform (Illumina, San Diego, CA, USA). Clean reads were aligned to the ‘Chiifu v4.0’ reference genome via HISAT2 [41,42], and read counts were calculated using HTseq [43]. Gene expression levels were analyzed by R v3.6.2 package “DESeq2” (1.10.1). DEGs were identified based on (1) adjusted *p* < 0.05, (2) Fragments Per Kilobase of transcript per Million mapped reads (FPKM) fold change ≥ 2.0.

### 4.5. Weighted Gene Co-Expression Network Analysis

Weighted gene co-expression network analysis was performed in R environment [44]. Based on the expression matrix (datExpr0), the Manhattan method was used to calculate the distance between samples, and average linkage hierarchical clustering was conducted to construct a sample dendrogram for outlier detection. Soft threshold power ranges from 1 to 10 and 12 to 20 were set, and the pickSoftThreshold function was applied to determine the optimal power value satisfying the scale-free network criterion (R^2^ > 0.85). Finally, a power of 9 was chosen to construct the weighted co-expression network.

The topological overlap matrix (TOM) was calculated, and 1−TOM was used as the dissimilarity metric (dissTOM) for gene clustering. Co-expression modules were identified using the dynamic tree cut algorithm. Module eigengenes (MEs), representing the first principal component of the module expression matrix, were calculated to characterize the overall expression pattern of each module. Pearson’s correlation analysis was performed to evaluate the relationships between modules and different treatments (vernalization duration or genotype), and a correlation heatmap was generated to visualize module–trait associations. Gene Ontology (GO) enrichment was performed using Tbtools (v2.466) [45]. The cut-off value for GO terms was *p*-value < 0.05. Modules significantly correlated with target traits were selected and imported into Cytoscape (v3.10.4) for network visualization and hub gene identification [46]. Function annotation of the DEGs was retrieved from known databases [47].

## 5. Conclusions

We systematically characterized bolting in non-heading Chinese cabbage using early- and late-bolting lines. By integrating phenotypic observations, SAM development, and transcriptome profiling, we found that in the early-bolting line, pre-bolting developmental stages are primarily regulated by gibberellin signaling. The expression of *XTH* genes is modulated by various plant hormones and closely linked to gibberellin and brassinosteroid signaling. These genes therefore act as critical regulators in meristem development and cell expansion [48]. The high expression of *XTH24* in ‘m662’ may promote cell wall reconstruction and further accelerate structural changes in the shoot apical meristem. The transcription factor *NAC062* (*BraA06g027310.4C*) is a member of the NAC family. Proteins of this family widely regulate plant development and stress responses. In the late-bolting line, vernalization cooperates with hormone signaling, cell cycle progression, and floral organ development to regulate bolting. The *BrFLC*-*BrSOC1* module plays a central role, with *BrFLC* repression and *BrSOC1* activation triggering bolting initiation. Multiple bolting-related hub genes were identified from co-expression modules, involved in cell wall remodeling, signal transduction, and SAM developmental reprogramming; their functions require further validation. This study delineates the dynamic transcriptional network governing early bolting, providing key candidate genes and a molecular framework for functional characterization and molecular breeding of non-heading Chinese cabbage.

## Data Availability

The raw data supporting the conclusions of this article will be made available by the authors on request.

## Supplementary Materials

The following supporting information can be downloaded at https://www.mdpi.com/article/10.3390/plants15131982/s1. Figure S1: Heatmap of correlation values among 45 transcriptome libraries; Figure S2: Weighted gene co-expression network analysis of relative differentially expressed genes in ‘m662’; Figure S3: GO enrichment analysis of key modules in ‘t151’; Figure S4: Weighted gene co-expression network analysis of differentially expressed genes in ‘t151’ with vernalization; Table S1: Primers for qRT-PCR; Table S2: Summary of transcriptome sequencing results after mapping; Table S3: Expression profiles of five time points’ genes; Table S4: GO enrichment terms of genes in MEpink module of ‘m662’; Table S5: GO enrichment terms of genes in MEbrown module of ‘m662’; Table S6: GO enrichment terms of genes in MEred module of ‘m662’; Table S7: GO enrichment terms of genes in MEturquoise module of ‘m662’; Table S8: GO enrichment terms of genes in MEgreen module of ‘t151’; Table S9: GO enrichment terms of genes in MEturquoise module of ‘t151’; Table S10: GO enrichment terms of genes in MEsalmon module of ‘t151’.
