# Supplementary material for: Dynamic Transcriptomic Networks Underlying Early Bolting in Non-Heading Chinese Cabbage
Source: Plants (Basel). 2026 Jun 26;15(13):1982. doi: 10.3390/plants15131982 (PMC13364084; doi:10.3390/plants15131982)
Supplement: Supplementary file 1 [file plants-15-01982-s001.zip › plants-4358370-supplementary/Supplementary Material/Supplementary_Material.pdf]

Supplementary Figures

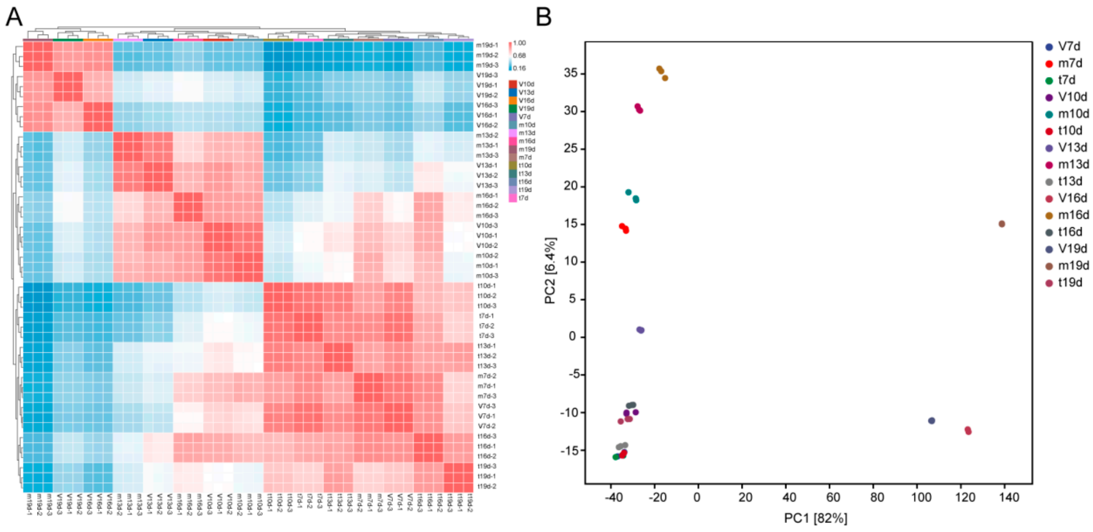

Figure S1. Relationships among transcriptome samples. (A) Heatmap of correlation values among 45 transcriptome libraries. (B) Principal component analysis based on all expressed genes. M stands for early-bolting line 'm662', t stands for late-bolting line 't151', V stands for 't151' with 10 days of vernalization.

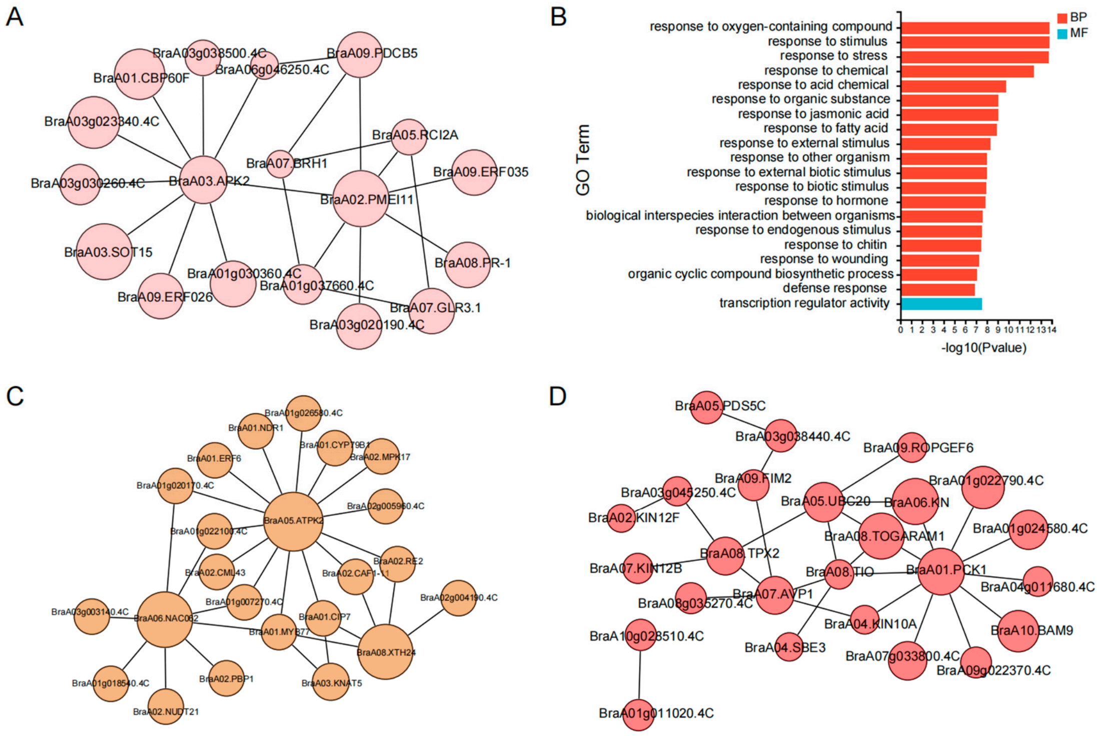

Figure S2. Weighted gene co-expression network analysis of relative differentially expressed genes in "m662". (A) Interaction network of the identified hub genes in

MEpink module, (B) GO enrichment of MEbrown module genes, (C) Interaction network of the identified hub genes in MEbrown module, (D) Interaction network of the identified hub genes in MEred module.

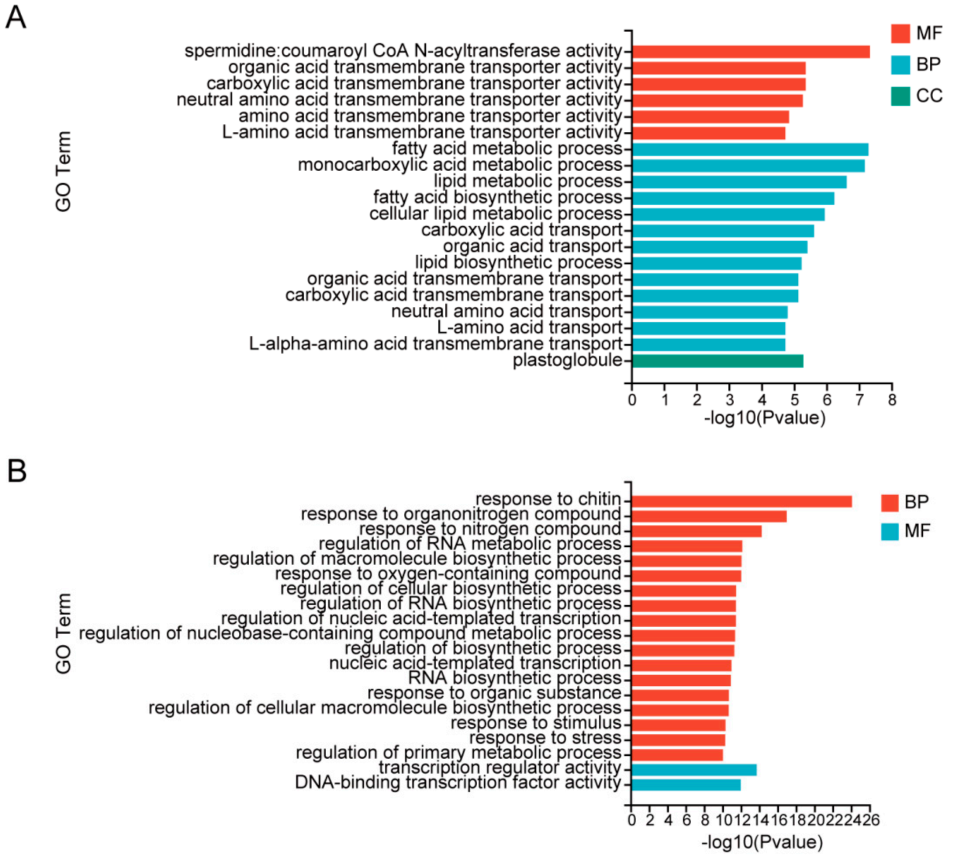

Figure S3. GO enrichment analysis of key modules in "t151". (A) GO enrichment of MEgreen module genes, (B) GO enrichment of MEred module genes.

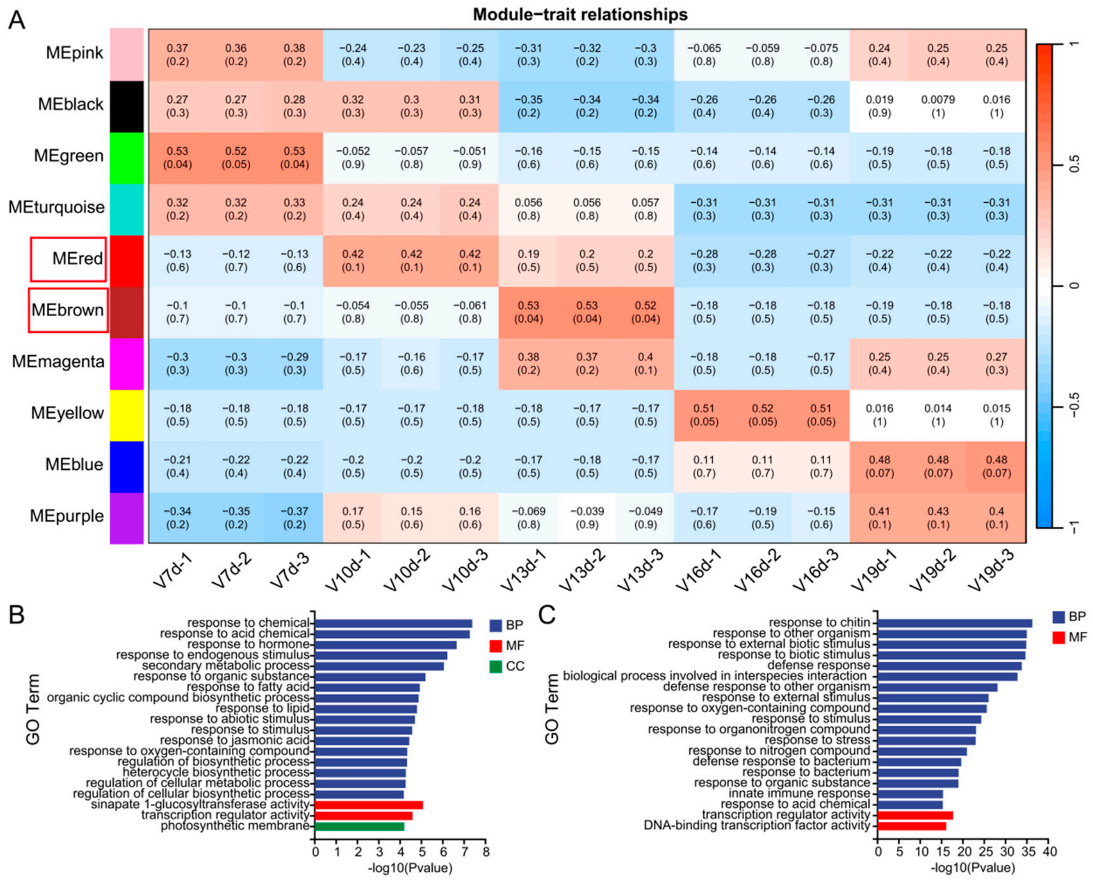

Figure S4. Weighted gene co-expression network analysis of differentially expressed genes in "t151" with vernalization. (A) Module − trait relationships. Red and blue indicate positive and negative correlations, respectively, with correlation coefficients and p-values shown. (B) GO enrichment analysis of genes in the MEred module. (C) GO enrichment analysis of genes in the MEbrown module.
